# The Tuberculosis Officer and His Work

**Published:** 1913-06

**Authors:** Latimer J. Short

**Affiliations:** County Tuberculosis Officer for Somerset; late Chief Tuberculosis Officer for Woolwich.


### THE TUBERCULOSIS OFFICER AND HIS WORK.

BY

LATIMER J. SHORT, M.D., B.S. Lond., M.D. Brist., D.P.H. Cantab., Fell. Roy. Inst. Pub. Health,

*County Tuberculosis Officer for Somerset; late Chief Tuberculosis Officer for Woolwich.*

---

THE tuberculosis officer is a comparative novelty. His immediate action and eventual position in medicine are as yet uncertain, and if the following description of him is somewhat diagrammatic, the writer pleads these circumstances as his excuse.

Two years' experience in city and county has indicated that the value of the tuberculosis officer's work will be in proportion to the extent to which he fits into his special place on the field.

He resembles the scrum half-back, and has to co-operate with nearly everybody in existence, if he is to be a permanent success. This alone makes it impossible to lay down any rigid boundaries, or even to give a fixed form to his actual administrative department; but the following details apply to a county which is just beginning to realise its immediate needs.

The county tuberculosis officer finds he has really to represent, and be able to supervise, the whole general scheme of tuberculosis work in his area. Prevention and treatment cannot, and certainly should not, be separated, and prolonged special and general clinical experience is not merely an essential but the *first* essential. Experience in treating tuberculosis in general private practice is one of the most important parts of a tuberculosis officer's education.

The coming of the National Insurance Act is merely a detail, and the tuberculosis officer must not let his work be cramped down to that of a mere insurance officer. The attack upon tuberculosis is a far more comprehensive movement, of which the Act supplies one particular only, and it was really in process of formation long before the Act was thought of.

Dealing first with the clinical side of his work, the tuberculosis officer has to advise the Insurance Committee as to the form of treatment which should be arranged for every patient who applies for "Sanatorium benefit." This misleading name includes all forms of benefit for all classes of tuberculous insured persons, even if it be a pair of blankets and a jar of malt extract. Obviously, he should meet the patient's own medical man when he has one, and talk over the whole matter with him, especially considering the patient's surroundings and facilities for home treatment. Unfortunately, in a scattered rural district it is often impossible for the tuberculosis officer to visit all the cases, or for the medical man to visit the central office, and correspondence has to replace the desired interview—a much less satisfactory method, especially when the tuberculosis officer has not yet received details of the patient's home conditions. In such cases I usually postpone the ordering of such extras as milk, bedding, an open-air shelter, etc., until more information is forthcoming.

Cases are also frequently referred to the tuberculosis officer by their medical attendant for help in diagnosis, either clinical or bacteriological, or for special tests, and these must be carefully investigated and reported on. School children form a large proportion of such cases, and others are sent for suggestions as to special treatment, especially where hospitals are few and far away.

On the *preventive* side, the tuberculosis officer must to a large extent be in charge of the details of the tuberculosis public health work in the whole of his area, under the general guidance of the county medical officer of health, and must be in constant touch with the tuberculosis health visitors and with the district medical officers, whose work he can materially lighten on one side even if he adds to it on others.

He must also be the connecting-link between the various agencies—professional and lay—now at work on the same campaign, which are really complemental to each other, if brought properly together, instead of being a mutual annoyance as so often happens at present. The securing of some material relief, an extra bedroom, temporary employment, and similar help, is often the most important part of the whole treatment of a case of tuberculosis. It is obvious that in a scattered county area these principles cannot be followed out without the provision of a large number of local centres in various parts of the county, where the tuberculosis officer can meet the patient and his doctor, can receive any sent to him for advice, can meet lay helpers, and can store such sundries as thermometers, sputum flasks, charts, cod liver oil and malt.

The local health visitor must make the same place her centre, keeping all her notes on cases and papers there for the information of the tuberculosis officer, to whom she reports weekly. It is convenient to call these " Dispensaries," although not much dispensing is done there, and they are the essential feature of a county scheme. There are already six in Somerset, and others are quickly to follow. They are all visited regularly at the same day and hour each week. It is at these " clinics " that cases for sanatorium beds are selected from among the many that one would like to get away from their homes. If a patient cannot come to the dispensary he is, as a general rule, unsuitable for a sanatorium at the time, but there is still grave difference of opinion among the profession as to what cases should and should not be sent to sanatoria.

The greater ease of getting patients away, owing to the Insurance Act, has resulted in persons being hurried off who are entirely incapable of benefiting themselves by the special treatment, and are merely a source of danger and disturbance to the really good cases at the sanatorium. A continuance of this may make the benefit an evil, and bring discredit upon the whole sanatorium scheme. If it is necessary, however, that the advanced and incurable should not be mixed with early cases, it is equally necessary that they should not be left to infect their own relatives at home, and the county proposes to establish camps of portable and cheap but cosy wooden buildings, preferably grouped round some small permanent building, for those in this condition. Some patients will not require to stay a great length of time, and this will redeem the place from the stigma of a "death hole"; but others will require treatment here for months or years until their death, and the provision of every extra comfort that can be obtained must make up for their compulsion.

The tuberculosis officer will soon have to face the question of tuberculous children, excluded from school for months or years, and steadily going to the dogs, body and soul, in sunless and airless courts and houses, with nothing to do, and no energy to do it. In an urban area, an open-air class or school is a cheap and eminently successful remedy. Anæmic and so-called "pre-tuberculous" children can be admitted and taught separately. It is only a partial provision, and in a scattered area it is obviously impossible, as these children cannot travel far, and a residential open-air school is the only thing. This should be centrally situated, and combine nursing and teaching, with a resident medical officer in charge. The tuberculosis officer should help to select cases for the school, many being originally referred to him by the school medical inspectors.

Experience has shown that children do not profit by institutional treatment, unless it is over six months in duration, and the normal stay at the open-air school should be one year, renewable if necessary. The actively tuberculous, in the infective stage, would be accommodated in a separate block, and would not mix with the others, schooling being inadvisable for them for the time being.

Recent investigations all go to show that pulmonary tuberculosis is far commoner in children than has hitherto been supposed, that its type and course differ from that seen in adults, and that its tendency to spontaneous apparent cure is very great, so that most of the cases are discovered only when active search is made for them. One says "apparent" cure because it is tempting to think that the great incidence of phthisis in the young adult is mostly but a recurrence of a previous, often unnoticed, infection in childhood.

This ushers in the vexed question of "contacts," by which one means the near relatives of a known case who are living with him in the house. It has been shown by Halliday Sutherland and others that there is a much greater incidence of tuberculosis in these than in people who are not thus described, owing to their close association with the sufferer, and it is only by a systematic and thorough clinical examination of these that the tuberculosis officer or anyone else can hope to get hold of the very earliest cases, before they are bad enough to seek medical advice on their own initiative.

In Woolwich, during 1911–12, out of 842 "contacts" examined clinically, I found no less than 291 who were either affected or so suspicious that immediate treatment was desirable, while as many as 63.5 per cent. of our patients (nearly 1,000 in number) apparently caught their disease from a previous case in the family and house. The difficulty is—"By whom should they be examined?" Members of a family are frequently under different doctors, and one could not properly examine the patients—either present or past—of a colleague.

In London general practice I found that any attempt to examine contacts was resented and suspected by the patient as an attempt to "make work," even if no fee were asked, and it was found better that an outside man should make the examination, and report to the practitioner attending the original case. After all, the only important thing is that the contacts should be thoroughly examined,and the tuberculosis officer is satisfied if he knows that each one can be accounted for, and that they are not being dropped between two or more stools.

Another very important branch of the work is the investigation and reform of the housing, sanitary, sleeping and economic arrangements, for bad housing means tuberculosis. This is women's work, and I have never found it really successfully accomplished by mere men, however tactful and earnest. It is perfectly true that many practitioners make a point of most carefully investigating these matters themselves, but it is also true that others cannot spare the time, or have not the inclination to do so, and this being so, it seems best for the health visitors to visit all cases—satisfactory and doubtful alike, if a really comprehensive scheme is to be followed. The tuberculosis officer does no visiting unless he receives a request from the medical attendant to do so, or in consultation with him, or on bedridden cases applying for "sanatorium benefit."

As to treatment by the tuberculosis officer, there will always be cases which cannot afford the long and continuous treatment that tuberculosis demands if success is to be attained, and for whom no other provision is made. These should be treated at the dispensaries, and their number will depend on what arrangements are made with the profession in the future for the further attack on this widespread disease. It would be a mistake to deprive the tuberculosis officer of all experience in treatment, apart from the question of expense, as he then gradually ceases to be a clinician, but co-operation with the general medical practitioner must be maintained—and should not be difficult to maintain—in this as in all other branches of the great scheme against tuberculosis.

The chief difficulty of the future will be to prevent the administrative side from swamping the clinical, for when the tuberculosis officer develops into an office machine, however efficient, his usefulness to medical science will be in very serious danger of coming to an end.

---

### THE THIRD SOUTH MIDLAND FIELD AMBULANCE.

